# Translational, Precision, and Personalized Medicine in Gastroenterology

**DOI:** 10.3390/ijms23158201

**Published:** 2022-07-25

**Authors:** Marcello Candelli

**Affiliations:** Emergency Medicine Department, Fondazione Policlinico Universitario Agostino Gemelli—IRCCS, Università Cattolica del Sacro Cuore di Roma, Largo A. Gemelli 8, 00168 Rome, Italy; marcello.candelli@policlinicogemelli.it

In recent decades, tremendous progress has been made in the medicinal field in understanding the molecular mechanisms underlying human pathologies, due to the significant development of advanced laboratory techniques and technologies. As a result, better knowledge of pathologies has also led to greater diagnostic, prognostic, and therapeutic opportunities. There have also been some important innovations in the field of gastrointestinal diseases, which are the focus of this Special Issue. In neoplastic diseases, for example, the development of immunotherapy and the genetic and molecular classification of various malignancies have led to significant advancements. Some are still being explored: the use of chimeric antigen receptor T-cell (CAR-T) and natural killers (CAR-NK), which have been very innovative in oncohematology, with six drugs being approved for the treatment of leukemia and myeloma [1], is still at an experimental stage in gastrointestinal diseases, although initial encouraging data are available from in vitro and animal models and from case series in humans (especially in metastatic colorectal cancer) [2,3,4]. In addition, genetic studies have shown that approximately 15% of colorectal neoplasms have a deficiency in the mismatch repair (MMR) pathway and microsatellite instability [5]. Initially, these alterations were associated with hereditary non-polyposis colorectal cancer (HNPCC), but it was later found that sporadic neoplasms may also have these mutations [6]. The defect led to a lack of DNA repair mechanisms and favored the appearance of new mutations, resulting in a high risk for the development of neoplasms [5]. Later, immunotherapy was found to be particularly effective in this subset of neoplasms because the high number of mutations led to the expression of a greater number of neoantigens, the increased accumulation of inflammatory cells, and the consequent locally increased release of cytokines [5]. The introduction of immunotherapy with checkpoint inhibitors of programmed death 1 (PD-1) is also capable of disrupting the mechanisms by which cancer cells evade the action of T cells and interrupting the processes that prevent programmed cell death [7]. For this reason, this class of drugs has been approved in the United States and, more recently, in Europe for the treatment of advanced colorectal neoplasms with MMR+. Finally, given the results achieved in endometrial tumors, dostarilimab, another PD-1 inhibitor, was successfully used in a phase II clinical trial in metastatic colorectal tumors that did not respond to other treatments, with a response achieved in 100% (12/12) of treated patients [8]. This is a clear success of translational medicine and personalized medicine. Promising therapies and accurate diagnostic methods are becoming more common in gastroenterology. For example, we see that there is a lot of interest in identifying the prognostic factors that can lead to identifying which IPMNs will develop into neoplasms. As we know, the only possible therapy for IPMN is surgery. However, duodenocephalopancreatectomy is not a simple procedure and is associated with significant mortality and morbidity rates, as well as a marked reduction in quality of life. For this reason, it is essential to identify the risk factors associated with cancer in order to only perform this procedure in individuals at highest risk [9]. Outside of oncology, two other major chapters have sparked a revolution in gastroenterology in recent decades: biological drugs and the interaction of the microbiota in inflammatory bowel disease. In particular, it now seems clear that the gut microbiota may play a fundamental role in both the development and progression of disease. Both endogenous and exogenous LPSs can both lead to increased intestinal permeability and promote inflammation at the local level, and modulation of the microbiome with probiotics and diet has been shown to be an effective treatment for inflammatory bowel disease (IBD) [10]. In contrast, the use of biologics, particularly anti-TNFα, has transformed the treatment of IBD. Unfortunately, few therapeutic strategies are available to gastroenterologists for corticosteroid- and anti-TNFα-resistant disease. In ulcerative colitis that does not respond to other treatments, Jak inhibitors have emerged as a very interesting option. About a quarter of patients respond satisfactorily to this strategy [11]. Currently, only one molecule is approved for clinical use, but several other molecules in the same class are close to market and may prove useful in the treatment of Crohn’s disease. Their particular properties (they are small molecules that are rapidly bioavailable, have a short duration of action, and are unlikely to trigger the mechanism of immune tolerance that occurs with other biologic drugs) and their ability to determine response by examining the phosphorylation of their target (STAT3) at the nuclear level, facilitating a rapid decrease in levels of IL-4, or identifying the hub gene make them promising candidates for personalized therapy in patients with ulcerative colitis [12,13]. 

Translational medicine, personalized medicine, and precision medicine are the future. We are taking the first steps in this direction by reaping the rewards of basic molecular research that will eventually lead to new therapies. However, the optimism of recent years should not obscure the fact that we are only at the beginning of this journey and that there is still much to be done and understood to best diagnose and treat gastrointestinal diseases. The future is now ours.
